# E-portfolio as an effective tool for improvement of practitioner nurses’ clinical competence

**DOI:** 10.1186/s12909-024-05092-z

**Published:** 2024-02-05

**Authors:** Nastaran Najaffard, Aeen Mohammadi, Rita Mojtahedzadeh, Afagh Zarei

**Affiliations:** 1 Department of E-learning in Medical Education, School of Medicine, Tehran University of Medical Sciences; and Clinical Nurse at Imam Sajjad Hospital, Tehran, Iran; 2 Department of E-Learning in Medical Education, Center of Excellence for E-learning in Medical Education, School of Medicine, Tehran University of Medical Sciences, No. 2, Dolatshahi Alley, Naderi St., Keshavarz BLVD, Tehran, Iran; 3 Education Development Center, Birjand University of Medical Sciences, Birjand, Iran

**Keywords:** Clinical governance, E-portfolio, Nursing, clinical competence

## Abstract

**Background:**

Clinical competence is essential for providing effective patient care. Clinical Governance (CG) is a framework for learning and assessing clinical competence. A portfolio is a work-placed-based tool for monitoring and reflecting on clinical practice. This study aimed to investigate the effect of using an e-portfolio on the practitioner nurses’ competence improvement through the CG framework.

**Methods:**

This was a quasi-experimental study with 30 nurses in each intervention and control group. After taking the pretests of knowledge and performance, the participants attended the in-person classes and received the educational materials around CG standards for four weeks. In addition, nurses in the intervention group received the links to their e-portfolios individually and filled them out. They reflected on their clinical practice and received feedback. Finally, nurses in both groups were taken the post-tests.

**Results:**

Comparing the pre-and post-test scores in each group indicated a significant increase in knowledge and performance scores. The post-test scores for knowledge and performance were significantly higher in the intervention group than in the control one, except for the initial patient assessment.

**Conclusion:**

This study showed that the e-portfolio is an effective tool for the improvement of the nurses’ awareness and performance in CG standards. Since the CG standards are closely related to clinical competencies, it is concluded that using portfolios effectively improves clinical competence in practitioner nurses.

**Supplementary Information:**

The online version contains supplementary material available at 10.1186/s12909-024-05092-z.

## Introduction

Clinical competence is essential for nurses to provide safe and effective patient care [1]. It refers to applying knowledge, attitude, skills, critical thinking, and decision-making in the clinical setting independently to provide optimal patient care. Clinical competence, which is the outcome of nursing education [2] has an association with other outcomes such as safety and satisfaction of patients, quality of care [3], low rate of job burnout [4], high-quality working life [5], self-efficacy, professional confidence, and effective use of clinical skills for nurses [6], which are crucial in the health care system.

Although different studies indicate that the clinical ability of nurses has different aspects such as professional responsibility, care management, interpersonal relationships and interprofessional care, and quality improvement [7], its goal, as mentioned earlier, is providing safe and effective patient care. Clinical competence develops during nursing education [8] and continues in the workplace [9]. Therefore, in addition to improving nursing students’ clinical competence [10, 11], we should also look for a framework to monitor and improve this competence in nurses working in clinical environments.

An accreditation framework that is used to evaluate and improve clinical performance is clinical governance (CG), which refers to the processes, systems, and structures that healthcare organizations use to ensure high-quality patient care [12]. It includes a quality assurance process that often leads to quality improvement activities [13]. CG standards cover items like prevention and health, care and treatment, management of nursing services, compliance with the rights of the service recipient, and drug and equipment management; which are closely related to the nursing staff duties. Hence, training nurses about CG standards is of great importance. CG provides a structure for clinical staff competence to be continually assessed and improved. It can be achieved through audit, feedback, and clinical supervision. The CG framework ensures continuous monitoring and evaluation of the quality of care provided by nurses and supports their ongoing professional development, which leads to delivering high-quality care to patients [14]. The guidelines for CG implementation in hospitals are scrutinized and set as standards. These standards cover not only the requirements of achieving clinical governance but also address local and context-based needs [15]. Clinical staff, including nurses, need to be familiar with these standards and become competent to apply them in practice [14]. So, there is a need to use appropriate learning and assessment tools in this regard.

A portfolio is one of the work-placed-based tools that can help nurses reflect, develop, and monitor their clinical competencies [16, 17]. As a structured, evidence-based tool, a portfolio shows the fulfillment of a profession’s standards for everyone practicing that career. It includes their vision of future growth and development [18] and provides the chance for capacity building in nursing continuous education [19] through reflecting on the practice and comparing the skills with the standards set by regulatory bodies. It offers the opportunity for self-assessment, identification of learning needs, and development of learning plans [20]. Thus, the portfolio can be used for learning and evaluation purposes in nursing [21].

A portfolio can be delivered either paper-based or electronically. Electronic portfolios (e-portfolios) have several advantages over paper-based ones, including more accessibility by many learners, easier shareability and storage, more portability and transferability, enhanced dynamicity, the possibility of uploading multimedia, and the chance of more learner expression [17, 22]. Despite these advantages, an e-portfolio is a relatively new tool for nursing education, and there is a need for further evaluation of its usage in clinical practice [16]. Furthermore, learners’ computer literacy level and access to the appropriate devices and the Internet are challenges for implementing e-portfolios [17, 19, 23]. Therefore, it is important to implement a user-friendly and accessible tool for designing the e-portfolio. Google Docs tool can be a suitable platform for this purpose, because of its availability and compatibility to different devices.

Finally, a scoping review, published in 2022, gathered evidence on the role of e-portfolios in scaffolding learning in healthcare disciplines including nursing. The authors recommend that there is a need to conduct further interventional and longitudinal studies, especially in continuous professional development after graduation. Assessing measurable learning outcomes with a focus on specific competencies helps to enrich the literature about the impact of e-portfolios on clinical performance. Also, optimizing e-portfolio development to guarantee its accessibility and user-friendliness is suggested [24]. These recommendations are aligned with providing evidence to use an e-portfolio to overcome some barriers to enhance the clinical competencies of nursing staff, which is the aim of CG implementation, like training being relevant to real work needs [11, 14], delivering high-quality training programs, learning through real clinical practice [14], covering the gap between the knowledge and clinical practice, and lack of feedback to clinical performance [11].

Therefore, considering (a) the importance of fostering nurses’ clinical competence, (b) the alignment of implementing the CG standards with this purpose, (c) the role of nurse training in achieving these standards, (d) the need to apply appropriate user-friendly and accessible learning and assessment tools, and (e) the need to assess the role of e-portfolio as a learning tool in acquiring clinical competencies; this study aimed to investigate the effect of using an e-portfolio on the nurses’ knowledge and clinical competence. We believe that the results of this study would add new perspectives to the literature to implement such easy-to-use educational strategies in clinical training.

## Methods

This was a quasi-experimental study with a pretest-posttest non-equivalent control group design performed in Imam Sajjad Shahriar General Hospital of Tehran University of Medical Sciences from September to November 2021. The nurses of this hospital needed to participate in Continuous Professional Development (CPD) programs for their annual promotion. The hospital’s Clinical Governance Committee (CGC) decided to include training courses about CG in their CPD agenda; so that the nurses would have enough incentive to participate in them. We selected two Internal Medicine wards with 34 and 37 nurses and randomly assigned them to the intervention and control groups. These two wards had a duration of patients’ hospitalization of over 12 h.

The inclusion criterion for nurses was having at least two years of work experience in the Internal Medicine ward. Finally, 30 nurses from each ward could participate in the study.

The Ethics Committee of Tehran University of Medical Sciences approved the study (reference code: IR.TUMS. VCR.REC.1399.379).

### Creating the contents

We used the Iranian GC standards as the framework for learning and reflecting. The CGC devised the educational objectives required for nurses to learn about CG based on the related national standards. These standards were classified into “initial patient assessment,” “surgical and anesthesia care,” “prevention and control of infection,” “drug management,” “laboratory and blood transfusion services,” and “service recipient support.” To cover the standards and their related learning objectives, we developed a 71-page booklet, six podcasts (a total of 112 min), and a 20-minute multimedia e-content to be delivered to both control and intervention groups. The CGC confirmed the contents’ coverage of the intended objectives.

### Study instruments

We used two instruments to assess the participants’ knowledge and practice before and after the intervention in both study groups. To investigate the knowledge, we used an electronic quiz consisting of 40 multiple-choice questions with a maximum score of 40 which was devised based on the learning objectives. Three experts in the field and two medical educationalists validated the questions ensuring their appropriate coverage of the learning objectives and correctness. Additionally, the test’s internal consistency of 0.79 was calculated using the Kuder-Richardson Formula 20. Appendix 1 includes some sample questions of the knowledge test.

Furthermore, we assessed participants’ performance using six checklists with a total of 100 items that were related to CG standards’ categories. Table 1 shows the checklists alongside their number of items and sample covered duties and tasks. The performance in each item was scored as “appropriate,” “inappropriate,” and “not performed,” with a maximum score of 100. Five CGC members and two medical educationalists approved the validity of the checklists.

**Table 1:** The checklists alongside their number of items and sample covered duties and tasks for assessing the participants’ performance in clinical governance standards in the study groups

No.	Category of clinical governance standards	No. of items	The sample covered duties and tasks
1	Initial patient assessment	26	Gathering and recording the patient’s necessary information; checking the state of consciousness; appropriate history taking; assessing the patient’s signs and symptoms, disabilities, possibility of fainting, and bedsores; nutritional assessment; and recording the nursing diagnosis
2	Surgical and anesthesia care	14	Doing the nursing duties of pre and post-operation like patient authentication, checking the patient being NPO, shaving the operation site …; checking the state of consciousness; examining the operation site status; and monitoring oxygen therapy
3	Prevention and control of infection	26	Correct usage of personal protective equipment, correct hand washing or rubbing, using disinfection solution, correct disposal of sharp objects, following sterilization protocols and not using jewelry and nail polish while conducting procedures
4	Drug management	14	Correct drug administration and injection, paying attention to drug side effects and reactions according to the patient’s signs and symptoms
5	Laboratory and blood transfusion services	10	Gathering laboratory samples like urine and sputum, correct blood products transfusion, and monitoring the patient during transfusion
6	Service recipient support	10	Introducing oneself to the patient, briefing the patient about the environment and treatment process, obtaining informed consent, using appropriate uniforms, and following etiquette

### Conducting the intervention

The study duration was six weeks for each of the control and intervention groups, including one week for taking the pre-tests, four weeks for the instruction, and one week for getting the post-tests. Four weeks seemed to be a reasonable time for nurses to apply all CG standards to patient care. To avoid contamination, we first conducted the study in the control group. Moreover, there was a long distance between the two wards included in the study on the hospital campus, and their facilities like the pavilion and restaurant were separate. Meanwhile, for more certainty, the questions were loaded one by one on each web page and the test links were deactivated for each participant immediately after submitting the test. To minimize the pre-test effect, the sequence of the questions and items and the wording were different between the pre and post-tests, though the questions were the same. Also, a six-week interval between the pre and post-tests seems to be adequate for not recalling the questions.

The conducted steps in the control group were as follows: The participants signed the informed consent form and then took part in the knowledge test, which we delivered electronically through the hospital’s Learning Management System (LMS). In addition, two trained supervisor nurses referred to the ward together and filled out the performance checklists for each participant in the real work setting within a week. They assessed nurses’ performance either based on the records or by observing them while performing duties or tasks. They would discuss any inconsistencies in filling out the checklists until they reached a consensus.

After obtaining the pre-tests, the participants took part in three one-hour face-to-face classes taught by supervisor nurses and CGC members. These classes were delivered through an interactive lecture teaching strategy and were repeated three times so that the nurses could participate in them at their convenience. In addition, the nurses received the above-mentioned educational contents via the hospital’s LMS. This training was conducted for four weeks during which reminder messages were sent to the nurses every week to encourage them to review the taught materials and refer to the educational contents. Finally, the post-tests were obtained just the same as the pre-tests within the last week.

In the intervention group, the participants underwent the same steps as the control group. The only difference was receiving an e-portfolio besides the above-mentioned training, in which they could reflect on their performance and receive feedback. We devised the e-portfolio using the Google Docs tool. In this e-portfolio, indicators of each standard devised by the CGC were included. Table 2 shows a sample of the e-portfolio questions designed for the indicator of “patient authentication before surgery” related to the “surgical and anesthesia care” category of CG standards. The nurses were asked every week to first self-assess their performance based on a 5-point Likert scale (ranging from very good to very poor); second, reflect on their performance in the assessment week, and write the points of strengths and weaknesses (with a focus on identifying the missed tasks); third, reflect on their performance in comparison with the previous week(s); and fourth, mention their educational needs for further learning. At the end of each week, a trained supervisor nurse provided feedback to each nurse, trying to guide them to improve the quality of care provision. The nurses performed this reflection and feedback cycle for four weeks.

**Table 2:** A sample of the e-portfolio questions designed for the indicator of “patient authentication before surgery” related to the “surgical and anesthesia care” category of clinical governance standards

E-portfolio for clinical governance standards
Week no: …
1. How do you rate your performance in the “patient authentication before surgery” indicator this week? (Scale: very good = 5, good = 4, average = 3, poor = 2, and very poor = 1)
2. Reflect on your performance. Determine the related tasks or duties that you have or have not done about the above indicator. Points of strength/ performed tasks: … Points of weakness/ missed tasks: …
3. Reflect on your performance in comparison with the previous week(s).
4. Do you need training for performing the above indicator? What are your educational needs?
Supervisor nurse feedback based on the “Stop”, “Keep”, and “Start” (SKS) model (please do not write anything in this box)

**Fig. 1:**
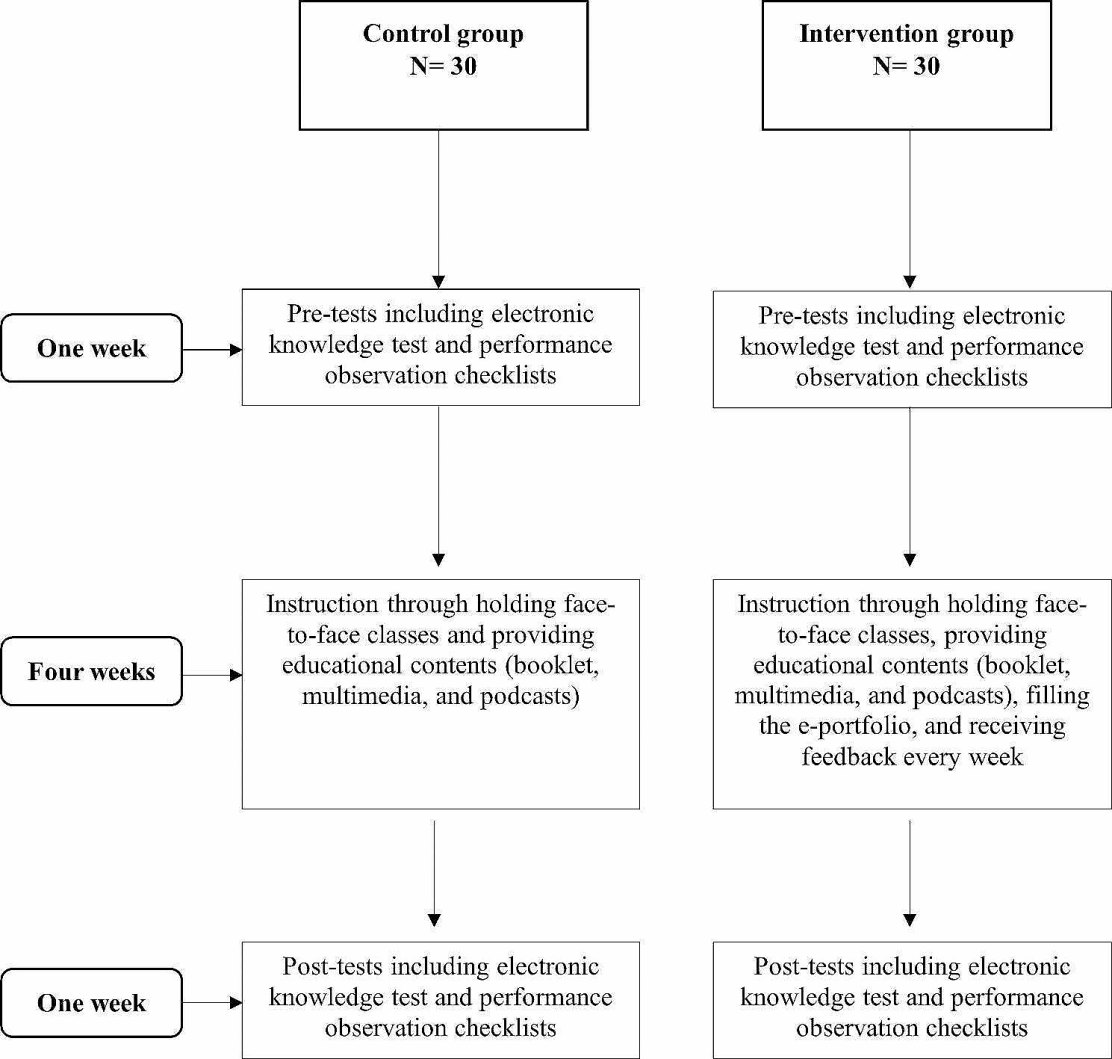
The schematic steps of the study in the intervention and control groups

The approach to providing feedback was based on the “Stop”, “Keep”, and “Start” (SKS) model, which promotes the active and regular solicitation of feedback and is useful for encouraging in-depth reflection [25]. In this model, the participants received feedback with a focus on three questions; “what should they start doing?”, “what should they keep doing?”, and “what should they stop doing? Hence, they had the chance to contemplate their behaviors, skills, and choices [26]. The summary of the study steps is illustrated in Fig. 1.

### Data analysis

IBM SPSS Statistics for Windows, Version 21.0., was used to analyze the data using independent and paired t-tests to compare the mean scores. Also, ANCOVA was used to eliminate the effect of confounding factors.

## Results

All 60 nurses participated in the study from the beginning to the end. There were no significant differences between the two groups concerning gender and years of nursing experience (Table 3).

**Table 3:** Comparison of the participants’ gender and years of nursing experience between the study groups

Variable		Control group		Intervention group		Sig.
		Number	Percent	Number	Percent	
Gender	Female	22	73.3	27	90	0.09*
	Male	8	26.7	3	10	
Years of experience		**Mean**	**SD**	**Mean**	**SD**	0.12**
		12.2	3.24	14.9	2.70	

* Chi-Square test, ** Independent t-test

The shape of the curve and Shapiro Wilk test confirmed the normality of all the knowledge and performance scores, so we used parametric tests for data analysis. No significant differences existed in pretest mean scores for knowledge and performance (*P*-values of 0.71 and 0.14, respectively). Within-group comparison of pre-and post-test scores indicated the significant achievement in knowledge and performance in both groups (*P*-values = 0.00). Finally, comparing the post-test scores between the intervention and control groups showed a significant difference in knowledge scores (*P*-value = 0.00) and no significant difference in performance scores (*P*-value = 0.16) (Table 4).

**Table 4:** Comparison of the knowledge and performance scores between the study groups

Variable	Group	Pre-test		Post-test		Sig*
		Mean	Standard Deviation	Mean	Standard Deviation	
Knowledge	Control	23.0	1.58	31.6	2.94	0.00
	Intervention	22.8	2.54	37.7	1.72	0.00
	Sig**	0.71		0.00		
Performance	Control	50.5	19.14	59.0	17.61	0.00
	Intervention	43.9	15.18	64.8	13.78	0.00
	Sig**	0.14		0.16		

*Paired t-test, **Independent t-test

To eliminate the effect of the pretest, we used ANCOVA, considering the pretest scores as the covariate, post-test scores as the dependent variable, and the groups (intervention and control) as a factor. The results showed a significant difference between the post-test scores for knowledge and performance (*P*-values = 0.00) (Table 5).

**Table 5:** Comparison of the post-test scores for knowledge and performance between the study groups considering the pre-test as a covariate

Source	Type III Sum of Squares	df	F	Sig.*
Knowledge	579.471	1	104.202	0.00
Performance	1777.336	1	30.288	0.00

* Analysis of Covariance

In addition, we compared the mean scores of the performance post-tests of different categories of CG standards between the two groups. There were significant improvements in post-test scores in the intervention group in all categories except for the initial patient assessment (Table 6).

**Table 6:** Comparison of the performance scores for different categories of CG standards between the study groups considering the pre-test as a covariate

Source	Group	Post-test scores		Type III Sum of Squares	df	F	Sig.*
		Mean	Standard Deviation				
Initial patient assessment	Control	9.2	5.46	25.796	1	3.722	0.05
	Intervention	10.2	5.5				
Surgical and anesthesia care	Control	12.8	4.43	2.458	1	0.384	0.00
	Intervention	14.8	3.94				
Prevention and control of infection	Control	9.0	3.61	87.599	1	31.671	0.00
	Intervention	10.7	2.83				
Drug management	Control	8.3	2.39	18.755	1	4.727	0.03
	Intervention	9.9	2.57				
Laboratory and blood transfusion services	Control	8.7	4.37	162.267	1	23.516	0.00
	Intervention	11.4	3.41				
Service recipient support	Control	9.0	3.05	51.454	1	8.805	0.00
	Intervention	9.7	3.0				

* Analysis of Covariance

## Discussion

In this study, two groups of nurses participated in the lecture-based classes and received the educational content. Meanwhile, nurses in the intervention group had to fill out an e-portfolio that aimed at reflecting on their clinical competence. The results showed significant increases in both the knowledge and performance scores in the intervention group compared to the control one.

The findings of the present research are in alliance with other studies like the ones of Bangalan [27], Lai [28], and Tsai [29], who confirmed the positive effects of e-portfolios on the knowledge and practice of learners. However, according to Lai [28], although an e-portfolio made progress in the theory and practice of nursing students, some occasional student stress was reported because of technical challenges. That was the reason for choosing the easy-to-use Google Docs tool for designing the e-portfolio in this study. We estimated that only some participants would be able to use more complicated software because of their computer literacy or device issues. As a result of implementing such an easy tool, participants of this study had minimal technical problems while using the e-portfolio.

Furthermore, we found a study in which no specific positive effect was observed for using an e-portfolio in a non-nursing context. Safdari and Torabi [30] implemented an e-portfolio as a formative assessment tool to improve the English writing skills of their learners and found no significant effect. The reason for that is more emphasis on self-assessment in their study, which is a must-to-be part of any portfolio. In contrast, continuous self-assessment was considered a key component in the present study. The nurses not only had to reflect on their performance weekly but also had the opportunity to compare their reflections with the previous week(s). This continuous self-assessment helped the nurses learn through personal reflections on their practice, resulting in self-estimation of the level of knowledge, skill, and understanding, as it is focused on in the literature [31]. To do so, the design for the e-portfolio in this study followed the recommended structure by Cope & Murray [17], including reflection on recent experiences; clarification of positive and negative aspects; self-identification of strength points and areas in need of further improvement; and notification of raising potential learning opportunities.

In this study, the e-portfolio effectively improved the nurses’ performance in all areas of CG except for the initial patient assessment. The participants had to assess different patient problems. Being able to solve one problem successfully is not a good predictor of the capability for other conditions [32]. So, initial patient assessment is case-specific, and nurses may need more encounters to fulfill this standard.

Although health professionals must be trained for clinical competencies during college or in actual practice, there are some concerns. For instance, more financial resources may limit the delivery of high-quality training programs [14]. Our experience indicates that an e-portfolio, which does not need so many financial resources, may solve this challenge.

We experienced some of the advantages mentioned in the literature for e-portfolios, which helped the intervention group acquire higher knowledge and better performance. Like the Hoveyzian study [21], the present e-portfolio, as a learning tool, provided the chance to link theory and practice, and nurses became aware of their strengths and weaknesses through reflection and with the help of provided feedback. In addition, just as in the experience of Pennbrant [33], learning through the e-portfolio method could help nurses understand the expectations in their practice.

This study had some points of strength and limitations. Considering the positive results of this study and the advantages of the e-portfolio, this strategy can be adopted in other hospitals and clinical environments. The barrier of providing appropriate software for e-portfolio development can be overcome using the free Google Docs facility, as implemented in our experience. In addition, we performed a real workplace assessment by observing the nurses’ performance according to the validated checklists, which makes the results more applicable. Despite these advantages, we had no possibility for randomization of the participants between the control and intervention groups, which could result in the same limitations of quasi-experimental studies. In addition, although we used some strategies mentioned before to avoid the contamination bias, we could not eliminate the risk. Other potential limitations are the short duration of the instruction (four weeks) in each group and the possibility of pre-test bias in the case of nurses remembering the questions of the knowledge test and the items of the checklists.

We suggest longer interventions with more self-paced usage of e-portfolios. Also, further studies in different contexts and other populations are recommended. Conducting studies to understand users’ experiences of such tools explores their being truly user-friendly, accessible, and efficient for individuals. Also, examining the long-term impact of using e-portfolios on professional growth, skill development, and clinical performance may be useful.

## Conclusion

Considering the importance of clinical competence in improving the quality of patient care, implementing appropriate training strategies to enhance nurses’ competencies is necessary. Based on the findings of this study, the Google Docs-based e-portfolio contributed to enhancing the overall clinical competence of the nurses in various aspects. So, we recommend using an e-portfolio as a learning tool for improving nurses’ clinical performance. Meanwhile, if it is not possible to provide professional e-portfolio software, accessible and user-friendly tools like Google Docs can be customized to serve as the infrastructure.

### Electronic supplementary material

Below is the link to the electronic supplementary material.

Supplementary Material 1

## Data Availability

The datasets used and/or analyzed during the current study are available from the corresponding author upon reasonable request.
