# Supplementary material for: E-portfolio as an effective tool for improvement of practitioner nurses’ clinical competence
Source: BMC Med Educ. 2024 Feb 5;24:114. doi: 10.1186/s12909-024-05092-z (PMC10845774; doi:10.1186/s12909-024-05092-z)
Supplement: Supplementary file 1 — Supplementary Material 1 [file 12909_2024_5092_MOESM1_ESM.pdf]

Article title:
E-portfolio as an Effective Tool for improvement of Practitioner Nurses' Clinical Competence

Appendix 1. Sample questions of the test for assessing the nurses' knowledge about clinical governance standards

1. What is the most influential factor for patient safety?
   a) Correct patient authentication
   b) Effective communication
   c) Correct history taking
   d) The physical environment of the ward

2. If the patient is not conscious, how do you match her/his information with the bracelet?
   a) Matching with the information written above the bed
   b) No need to match the information with the bracelet
   c) Asking parents or accompanying person
   d) Checking the information with the previous shift nurse

3. What is the best technique used in patient delivery?
   a) SBAR
   b) IS(O)BR
   c) Read back
   d) Oral

4. How many times a day can chemical restraint be implemented with a doctor's prescription as Stat or PRN?
   a) Five times
   b) Four times
   c) Three times
   d) There is no limit

5. Who is responsible to provide patient self-care training to prevent the risk of bed sores and falls in the inpatient wards?
   a) The nurse responsible for the patient
   b) Head nurse of the ward
   c) Physician
   d) Rehabilitation expert

6. Which of the followings **is not** correct regarding the transfer and delivery of the patient from the ward to the operating room?
   a) The surgical team confirms the patient's written informed consent.
   b) The patient's fasting before the operation is in accordance with the doctor's order and the timing of the operation.

c) The patient can be transported to the operating room only with a wheelchair or stretcher.
d) The patient cannot wear artificial nails or jewelry.

7. How many seconds does the handshake last?
   a) 30 to 40
   b) 20 to 30
   c) 40 to 60
   d) 30 to 50

8. Who administers anesthesia and sedation to patients in the imaging unit?
   a) Responsible nurse
   b) Physician
   c) Anesthesiologist
   d) Anesthesia technician
